# The Effect of the Combined Action of Roscovitine and Paclitaxel on the Apoptotic and Cell Cycle Regulatory Mechanisms in Colon and Anaplastic Thyroid Cancer Cells 

**DOI:** 10.5402/2012/826305

**Published:** 2012-08-30

**Authors:** V. V. Pushkarev, O. I. Kovzun, V. M. Pushkarev, M. D. Tronko

**Affiliations:** Department of Fundamental Problems of Endocrinology, State Institution “V.P. Komisarenko Institute of Endocrinology and Metabolism”, AMS of Ukraine, Kyiv 04114, Ukraine

## Abstract

*Aim*. To study the significance of cyclin-dependent kinases (Cdks) in paclitaxel-dependent apoptosis in colon and undifferentiated thyroid cancer cells. *Materials and Methods*. Experiments were performed on undifferentiated thyroid carcinoma (KTC-2) and colon carcinoma (ARO) cell lines. Cells were treated with paclitaxel (Ptx) and inhibitor of Cdk, roscovitine. Cell survival test and Western blotting were used for characterization of the effects of paclitaxel and roscovitine on cancer cells. *Results*. It was shown that not c-Jun N-terminal kinase, but cyclin-dependent kinases are responsible for antiapoptotic Bcl-2 phosphorylation. Cdk inhibition enhanced the cytotoxic effects of Ptx at low drug concentrations. There was antagonism between Ptx and roscovitine at higher (25 nM) paclitaxel concentrations. *Conclusion*. Using of paclitaxel at low (2.5 to 5 nM) concentrations and roscovitine is a promising combination for further preclinical trials for the development of new therapeutic approaches to the treatment of colon and anaplastic thyroid cancer.

## 1. Introduction 

Compounds that stabilize microtubules (MSA), which include taxanes (paclitaxel, docetaxel), are effective anticancer drugs. It is known that the therapeutic effect of these drugs is associated with cell cycle arrest, followed by initiation of apoptotic processes [1]. However, the exact mechanism that links cell division impairment with apoptosis induced by drugs is still poorly understood. 

The mammalian cell cycle is controlled by cyclin-dependent kinases, whose activity is modulated by several activators and inhibitors [2]. Cdks are serine/threonine kinases that play a key role in regulating both cell cycle and transcription through the phosphorylation of transcription factors and tumor suppressor proteins involved in DNA replication and cell division [2].

The Cdk modulators include a 2, 6, 9-substituted purine analogue, roscovitine (CYC202), which inhibits Cdk activity directly by competing for the ATP-binding sites of Cdk and causing apoptosis within various tumor cells. A study of clinical utility of roscovitine showed its anticancer effects and limited toxicity for humans in clinical trials [3].

The aim of this study was to establish a connection between the effect of paclitaxel on cell cycle and induction of apoptotic processes in colon (CC) and anaplastic thyroid cancer (ATC) cell lines ARO and KTC-2.

## 2. Materials and Methods

### 2.1. Cell Lines and Conditions of Culturing

ATC cell lines KTC-2 were established at Kawasaki Medical School (Okayama, Japan). Human cancer cell line ARO (initially assumed to be anaplastic thyroid carcinoma cell line but recently reclassified into colon carcinoma) was initially provided by J. A. Fagin (University of Cincinnati College of Medicine, Cincinnati, OH, USA). 

Throughout all experiments, cancer cell lines were grown in RPMI 1640 supplemented with 5% fetal bovine serum (FBS) and 1% penicillin/streptomycin (all reagents from Invitrogen Life Technologies, Paisley, UK) in a 5% CO_2_ humidified atmosphere at 37°C. After 2 d incubation, when the culture reached about 80% confluence, cells were washed twice with PBS (pH 7.4) at 37°C, and a fresh medium was added to each dish. Cells were incubated for additional 24 h, exposed to the drug(s) as described below, and then collected at different time intervals.

### 2.2. Cell Survival Assay

Cultures were established in the 96-well flat-bottom microtiter plates in RPMI 1640 containing 5% FBS. Cell suspensions (100 *μ*L, *~*1000 cells/well) were added to each well and incubated for 24 h before treatment. Ptx (Wako Chemicals, Osaka, Japan) and roscovitine dissolved in dimethylsulfoxide (DMSO) and the control (DMSO only) were added to each well at varying concentrations, six wells for each concentration. After incubation, a water-soluble tetrazolium salt-based assay (WST) was performed as follows: 11 *μ*L of the cell counting kit solution (CCK-8, Dojin, Osaka, Japan) were added to each well and incubated for 1 h at 37°C. OD was read at 450 nm in a microplate reader.

### 2.3. Preparation of Cell Extracts

 Adherent cells were washed twice with an ice-cold PBS supplemented with sodium pyrophosphate and orthovanadate, scraped with a rubber policeman, collected in 1 mL PBS, and centrifuged for 3 min at 1000 rpm at 4°C. The pellet was then resuspended in 200 *μ*L of the lysis buffer (Cell Signaling Technology, Danvers, MA, USA) containing a cocktail of protease and phosphatase inhibitors. After 15 min on ice, lysates were centrifuged for 15 min at 15,000 g and stored at −80°C until use. Protein concentration was determined with a bicinchoninic acid assay reagent kit (Sigma, St. Louis, MO, USA) according to manufacturer's protocol. 

### 2.4. Western Blotting

Total cell lysates were boiled in a sample buffer (100 mM Tris-HCl, 4% sodium dodecyl sulfate, 0.2% bromophenol blue, 20% glycerol, 10% dithiothreitol) and separated by SDS-PAGE 7.5–15% gradient gels. The homogeneous 8 and 15% gels were used for better separation of high- and low-molecular-weight proteins, respectively. Forty micrograms of protein were applied per each lane. Proteins were transferred onto 0.2-*μ*m nitrocellulose membranes (Millipore Corp., Bedford, MA, USA) by semidry blotting. Membranes were blocked with tris-buffered saline/0.1% Tween 20 containing 5% nonfat dry milk or 5% BSA and incubated with primary antibodies (Cell Signaling Technology or Santa Cruz Biotechnology, Santa Cruz, CA, USA), as appropriate, at 4°C overnight. After washing three times with tris-buffered saline/0.1% Tween 20, the blots were incubated with horseradish peroxidase-conjugated species-specific secondary antibody (Cell Signaling Technology) for 1 h at ambient temperature and then again washed three times. Complexes were visualized using ECL reagents (Amersham, Arlington Heights, IL, USA).

### 2.5. Statistical Analysis

All data were expressed as a mean ± SD or mean ± SE. Differences between groups were assessed for statistical significance using Student's *t* test. *P* < 0.05 denoted the presence of a statistically significant difference.

## 3. Results and Discussion

**Figure 1:**
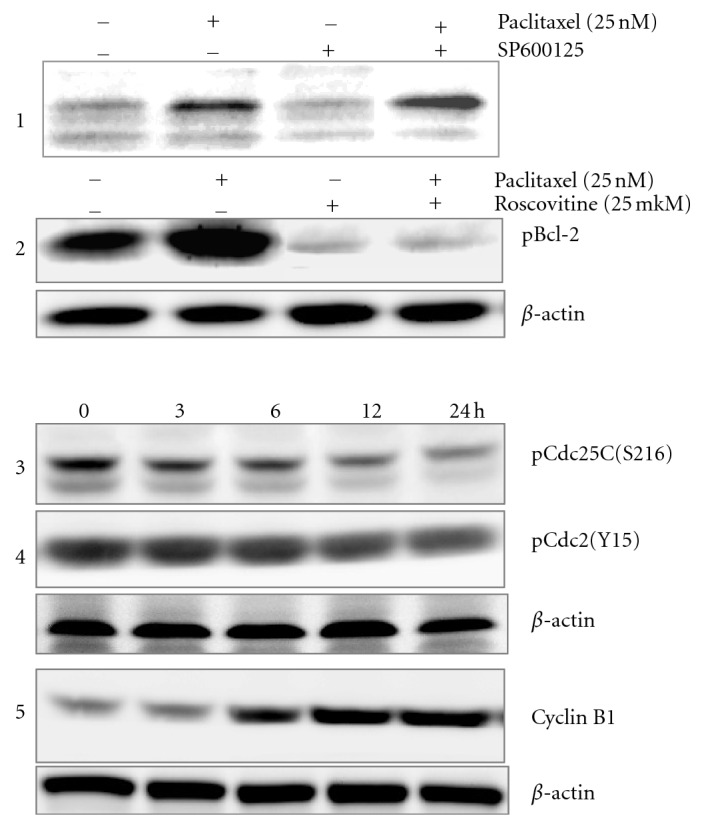
Effects of Ptx and roscovitine and combined effect of roscovitine + Ptx on phosphorylation of Bcl-2, activation and expression of proteins involved in regulation of cell cycle of KTC-2 cells.

It is believed that the basis of the mechanism of apoptosis induction by Ptx in tumor cell is phosphorylation with subsequent degradation of the antiapoptotic protein Bcl-2, which stabilizes the mitochondrial membrane [4, 5]. A decrease in this protein amount leads to an imbalance between pro- and antiapoptotic proteins causing a release of mitochondrial cytochrome *C* and other apoptotic factors followed by caspase-9 activation. The JNK-dependent phosphorylation of Bcl-2 and its further degradation were considered as the most probable event, which mediates the Ptx action [4]. However, experiment with a JNK inhibitor, SP600125, showed that inhibition of JNK in ATC cells did not reduce Bcl-2 phosphorylation as expected but even slightly increased it (Figure 1(1)). Since Ptx in ATC cells activates a number of cell cycle regulators [6], a specific inhibitor of Cdk, roscovitine, was used. It was found that inhibition of Cdk caused a nearly complete, qualitative inhibition of Bcl-2 phosphorylation (Figure 1(2)). Thus, Cdk activated by Ptx is directly or indirectly responsible for phosphorylation of antiapoptotic protein Bcl-2. These results are confirmed by data obtained on KB-3 human carcinoma cell line evidenced that Cdk1 phosphorylated Bcl-X_L_/Bcl-2 and thus attenuated their antiapoptotic function [7]. 

It should be noted that JNK, and to a lesser extent p38MAPK, also mediates Ptx-induced apoptosis in ATC cells [4] but obviously through parallel mechanisms. Future experiments will show whether there are any interrelationships between these mechanisms and Cdk or Cdk-dependent signaling. Some data point out the possibility of such an interaction [8, 9]. 

One of the roscovitine targets in the cell is Cdk1 (Cdc2) [10, 11], which in combination with cyclin B1 promotes the transition of cells from G2 phase to mitosis. It has been shown that in ATC cells Ptx, like other MSA, stopped the transition, caused an arrest of cycle at the G2/M stage [5]. This triggered the activation of a number of cell cycle regulators, which, on the background of a decrease in quantity of Cdk-inhibitors (p27^KIP1^ and p21^WAF1^), stimulated overcoming of the G2/M barrier [6]. Among the most important events, it should be noted the activation of phosphatase Cdc25C (dephosphorylation of ser216), which in turn activates Cdk1 by removing inhibitory phosphate at position 15 tyrosine residues, and a significant increase in Cdk1 cofactor cyclin B1 expression (Figures 1(3)–1(5)). Perhaps an excessive activation of Cdk1, which is observed under the influence of Ptx, is one of the triggers that initiated the process of apoptosis in tumor cells.

Thus, it may be suggested that the initiation of mitochondrial apoptosis in cells of anaplastic thyroid cancer under the action of Ptx is mediated by cyclin-dependent kinases.

**Figure 2:**
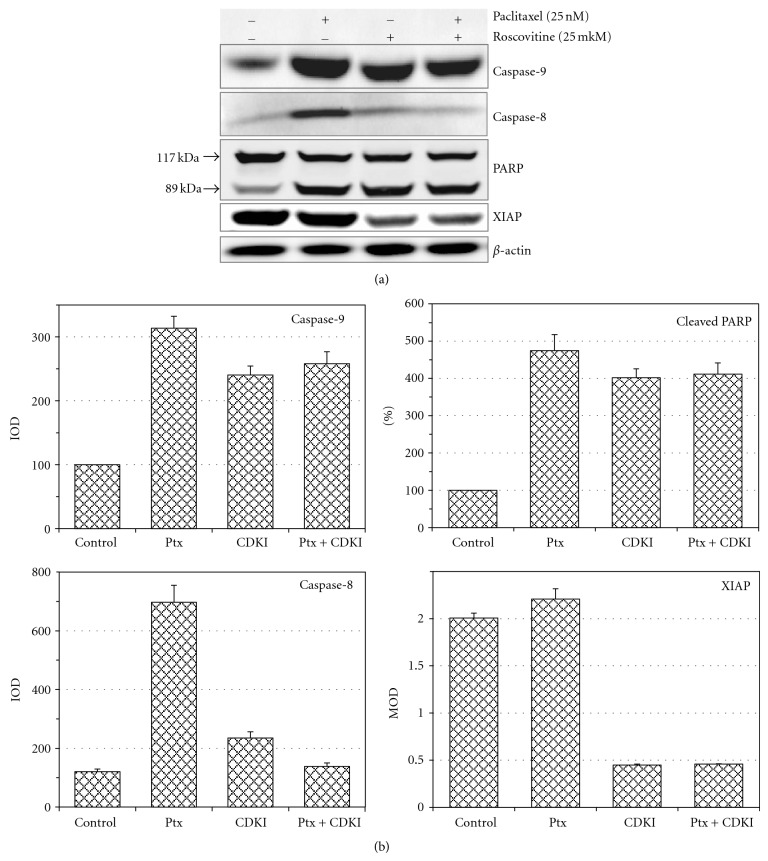
Effects of Ptx and roscovitine and combined effect of roscovitine + Ptx on activation and expression of proteins involved in apoptosis of KTC-2 cells. (a) Western blotting study. (b) Quantification of WB results. Data are mean of 4 OD measurements of one blot by scanning software ± SD value (error of scanning). CDKI: Cdk inhibitor, roscovitine; IOD: integrated OD, MOD: maximal OD of the bands.

However, a study of the effects of combined action of roscovitine and Ptx upon apoptotic mechanisms showed that roscovitine activated caspase-9, caspase-8, and PARP cleavage but attenuated Ptx-dependent activation of caspase-9, PARP cleavage, and especially caspase-8 activation (Figures 2(a) and 2(b)). On the other hand, roscovitine significantly decreased antiapoptotic XIAP level, which is high under Ptx action (Figure 2).

**Figure 3:**
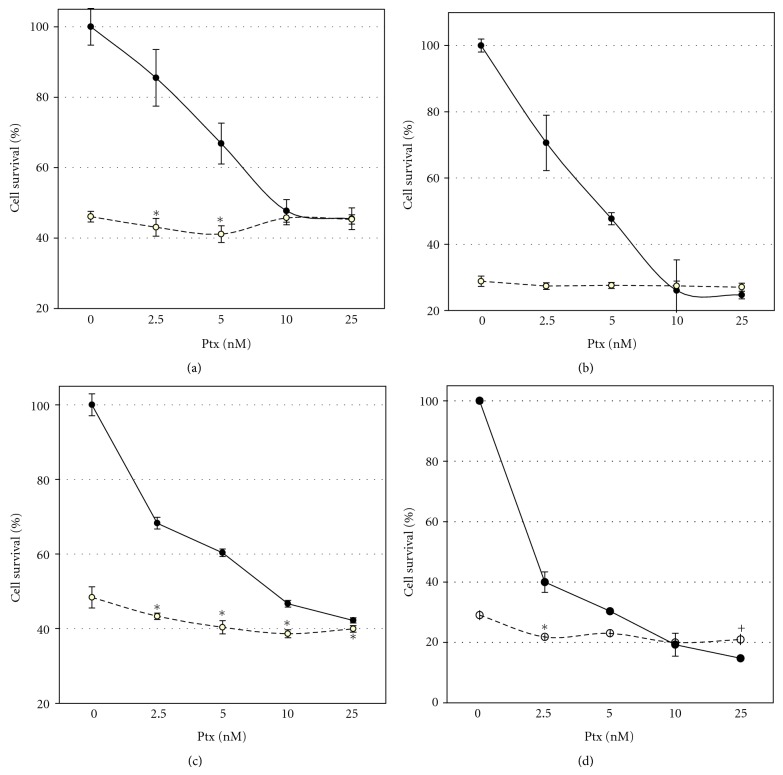
Effect of Ptx and roscovitine on cancer cell viability. (a, b) ARO cells; (c, d) KTC-2 cells. (a, c) 24 h of cell incubation with drugs, (b, d) 48 h. Data represent mean ± SD value (*n* = 6). ∗ represents significant difference from control (without Ptx); + represents that inhibition of Ptx effect by roscovitine is significant, *P* < 0.05 (pair Student's *t* test).

Cell survival study on ARO cells showed that after 24 h of incubation roscovitine enhanced Ptx cytotoxicity at low drug concentration, and there were no effects at higher Ptx concentrations: 10–25 nM and after 48 h of incubation (Figures 3(a) and 3(b)). In KTC-2 cells roscovitine, after 24 h of incubation, enhanced Ptx cytotoxicity at all studied drug concentrations (Figure 3(c)). After 48 h of incubation, roscovitine increased Ptx cytotoxicity at low (2.5–5 nM) concentrations and did not affect or even inhibited cell death at higher drug concentrations (Figure 3(d)). Certain inhibition of Ptx cytotoxicity at 25 nM by roscovitine probably reflected the reduced activity of caspases under combined effects of Ptx and roscovitine (Figures 2(a) and 2(b)). The difference in roscovitine action on ARO and KTC-2 cell line as well as a higher sensitivity of KTC-2 cells to the drugs may be due to the presence of an active *TP53* gene in the latter. It is known that roscovitine can induce activation and stabilization of p53 by suppression of MDM2 expression [12, 13]. 

Thus, roscovitine in ATC and CC cells showed marked proapoptotic effects, which can be explained by phosphorylation of antiapoptotic protein Bcl-2 and inhibition of IAP expression. There is evidence also that roscovitine suppressed antiapoptotic Mcl-1 expression and downregulated FLICE-inhibitory proteins in breast cancer cells [14]. Other data show that roscovitine increases the proapoptotic Bax and decreases antiapoptotic survivin and XIAP expression, resulting in caspase-dependent apoptosis of sarcoma cells [15]. 

Combined action of micromolar concentrations of roscovitine and low (1–5 nM) concentrations of Ptx may be a promising strategy for further preclinical investigations. 

## Abbreviations

Ptx: Paclitaxel
ATC: Anaplastic thyroid cancer
CC: Colon carcinoma
Cdk: Cyclin-dependent kinase
JNK: c-Jun N-terminal kinase/stress-activated protein kinase
FBS: Fetal bovine serum
BSA: Bovine serum albumin
PBS: Phosphate-buffered saline.

## References

[1] Bergstralh D. T., Ting J. P. Y. (2006). Microtubule stabilizing agents: their molecular signaling consequences and the potential for enhancement by drug combination. *Cancer Treatment Reviews*.

[2] Malumbres M., Barbacid M. (2009). Cell cycle, CDKs and cancer: a changing paradigm. *Nature Reviews Cancer*.

[3] Mohapatra S., Coppola D., Riker A. I., Pledger W. J. (2007). Roscovitine inhibits differentiation and invasion in a three-dimensional skin reconstruction model of metastatic melanoma. *Molecular Cancer Research*.

[4] Pushkarev V. M., Starenki D. V., Saenko V. A. (2004). Molecular mechanisms of the effects of low concentrations of taxol in anaplastic thyroid cancer cells. *Endocrinology*.

[5] McGrogan B. T., Gilmartin B., Carney D. N., McCann A. (2008). Taxanes, microtubules and chemoresistant breast cancer. *Biochimica et Biophysica Acta*.

[6] Pushkarev V. M., Starenki D. V., Saenko V. A. (2008). Differential effects of low and high doses of Taxol in anaplastic thyroid cancer cells: possible implication of the Pin1 prolyl isomerase. *Experimental Oncology*.

[7] Terrano D. T., Upreti M., Chambers T. C. (2010). Cyclin-dependent kinase 1-mediated Bcl-xL/Bcl-2 phosphorylation acts as a functional link coupling mitotic arrest and apoptosis. *Molecular and Cellular Biology*.

[8] Ghahremani M. H., Keramaris E., Shree T. (2002). Interaction of the c-Jun/JNK pathway and cyclin-dependent kinases in death of embryonic cortical neurons evoked by DNA damage. *The Journal of Biological Chemistry*.

[9] Du L., Lyle C. S., Obey T. B. (2004). Inhibition of cell proliferation and cell cycle progression by specific inhibition of basal JNK activity: evidence that mitotic bcl-2 phosphorylation is JNK-independent. *The Journal of Biological Chemistry*.

[10] Meijer L., Borgne A., Mulner O. (1997). Biochemical and cellular effects of roscovitine, a potent and selective inhibitor of the cyclin-dependent kinases cdc2, cdk2 and cdk5. *European Journal of Biochemistry*.

[11] Meijer L., Raymond E. (2003). Roscovitine and other purines as kinase inhibitors. From starfish oocytes to clinical trials. *Accounts of Chemical Research*.

[12] Lu W., Chen L., Peng Y., Chen J. (2001). Activation of p53 by roscovitine-mediated suppression of MDM2 expression. *Oncogene*.

[13] Dey A., Wong E. T., Cheok C. F., Tergaonkar V., Lane D. P. (2008). R-Roscovitine simultaneously targets both the p53 and NF-*κ*B pathways and causes potentiation of apoptosis: implications in cancer therapy. *Cell Death and Differentiation*.

[14] Ortiz-Ferrón G., Yerbes R., Eramo A., López-Pérez A. I., de Maria R., López-Rivas A. (2008). Roscovitine sensitizes breast cancer cells to TRAIL-induced apoptosis through a pleiotropic mechanism. *Cell Research*.

[15] Tirado O. M., Mateo-Lozano S., Notario V. (2005). Roscovitine is an effective inducer of apoptosis of Ewing's sarcoma family tumor cells in vitro and in vivo. *Cancer Research*.

